# Gynecologic–colorectal collaborative vaginal-side retrograde access to the rectovaginal space during laparoscopic posterior pelvic exenteration: a case report

**DOI:** 10.1097/RC9.0000000000001085

**Published:** 2026-10-09

**Authors:** Norihito Kamo, Momoka Ito, Keisuke Yoshida, Eisei Endo, Shinji Ohki, Ryuji Yamauchi

**Affiliations:** a Department of Obstetrics and Gynecology, Shirakawa Kosei General Hospital, Fukushima, Japan; b Department of Obstetrics and Gynecology, Fukushima Medical University School of Medicine, Fukushima, Japan; c Department of Surgery, Shirakawa Kosei General Hospital, Fukushima, Japan

**Keywords:** case report, fibrotic pelvis, gynecologic–colorectal collaboration, posterior pelvic exenteration, rectovaginal fistula, vaginal-side retrograde access

## Abstract

**Introduction and importance:**

Rectal cancer invading gynecologic organs, with a rectovaginal fistula and post-chemoradiotherapy fibrosis, is surgically challenging. Obliteration of the pouch of Douglas and the rectovaginal plane can render conventional posterior abdominal dissection unsafe.

**Presentation of case:**

An 85-year-old woman with cT4b rectal cancer suspected of invading the posterior vaginal wall and uterus developed a rectovaginal fistula after chemoradiotherapy. Despite marked tumor regression, severe fibrosis obliterated the pouch of Douglas and rectovaginal plane; vaginal narrowing and radiation-induced contracture limited transvaginal manipulation. During laparoscopic, bladder-preserving posterior pelvic exenteration, the gynecologic team performed a vaginal-side, retrograde resection of the posterior vaginal wall to obtain controlled access to the rectovaginal space. This enabled en bloc resection of the rectum, uterus, and posterior vaginal wall. Pathology showed residual adenocarcinoma confined to the rectal submucosa, grade 2 response, ypT1bN0, and no viable tumor cells in the vagina or uterus. Postoperative pelvic peritonitis resolved with antibiotics; no recurrence was observed at 10 months.

**Clinical discussion:**

This approach adapts a gynecologic vaginal-side retrograde access concept to colorectal surgery rather than using posterior colpotomy. It may enable controlled entry when posterior abdominal dissection is unsafe, although contamination, tumor spillage, and pelvic infection remain concerns in patients with a rectovaginal fistula.

**Conclusion:**

Gynecologic–colorectal collaborative vaginal-side retrograde access may be a practical option for laparoscopic posterior pelvic exenteration in selected patients with severe pelvic fibrosis. Further cases and longer follow-up are needed to assess oncologic safety.

## Introduction

Locally advanced rectal cancer involving adjacent gynecologic organs may require en bloc multivisceral resection or posterior pelvic exenteration to achieve an R0 margin, a key determinant of local control and long-term survival^[^1–8^]^. Surgical management becomes particularly challenging when rectovaginal fistula (RVF) and severe pelvic fibrosis develop after chemoradiotherapy^[^9^]^.

HIGHLIGHTS

- An 85-year-old woman with rectal cancer developed a rectovaginal fistula after chemoradiotherapy.
- Severe fibrosis obliterated the pouch of Douglas and the rectovaginal plane.
- Gynecologic–colorectal collaboration enabled vaginal-side retrograde access.
- The approach created a controlled entry point for laparoscopic en bloc resection.
- Its oncologic safety requires further evaluation with longer follow-up and in additional cases.

Post-treatment fibrosis may obliterate the Douglas pouch and the rectovaginal plane, making conventional abdominal posterior dissection hazardous. Forced dissection through a fused rectovaginal plane can increase the risk of bleeding, uncontrolled fistula disruption, pelvic contamination, and tumor violation^[^9–11^]^. In such cases, individualized access planning and close collaboration between colorectal and gynecologic surgeons is essential^[^9^]^.

Posterior colpotomy and retrograde vaginal approaches have been described in gynecologic surgery^[^12,13^]^. However, their application as an access strategy in colorectal surgery for a severely fibrotic pelvis remains less well established. Herein, we report a case of T4b rectal cancer with RVF in which a gynecologic–colorectal collaborative vaginal-side retrograde posterior vaginal wall resection was used to establish controlled access to the rectovaginal space during laparoscopic bladder-preserving posterior pelvic exenteration. This case report was prepared in accordance with the SCARE checklist^[^14^]^.

## Presentation of case

An 85-year-old woman, gravida 2, para 2, with a history of cerebral infarction, hypertension, type 2 diabetes mellitus, and two prior cesarean sections, presented with hematochezia. Her Eastern Cooperative Oncology Group performance status was zero. Colonoscopy revealed a circumferential tumor in the upper rectum, and biopsy confirmed adenocarcinoma (Supplemental Digital Content Figure S1a, available at: https://links.lww.com/IJSCR/A179). Pelvic examination revealed a firm mass involving the posterior vaginal wall, accompanied by stool-like vaginal discharge; however, a definitive fistula was not clearly identified at the initial evaluation. The carcinoembryonic antigen (CEA) level was 238.0 ng/mL.

Pelvic magnetic resonance imaging (MRI) suggested direct invasion of the posterior vaginal wall, with possible extension toward the uterus and lateral vaginal wall (Supplemental Digital Content Figure S1b, available at: https://links.lww.com/IJSCR/A179). The pretreatment clinical stage was cT4bN2aM0, cStage IIIC. The clinical timeline is summarized in Table 1. Following multidisciplinary discussion between colorectal and gynecologic surgeons, a double-barrel transverse colostomy was created. The patient subsequently received concurrent chemoradiotherapy with capecitabine (1800 mg/day) and pelvic irradiation (50.4 Gy in 28 fractions), followed by three cycles of capecitabine maintenance therapy. The CEA level decreased to 3.3 ng/mL.

**Table 1:** Clinical timeline from diagnosis to postoperative follow-up.

Time point	Clinical event/findings
Initial presentation	Hematochezia; colonoscopy revealed a circumferential rectal tumor. Biopsy confirmed adenocarcinoma.
Initial pelvic evaluation	Posterior vaginal wall mass and stool-like vaginal discharge; RVF was suspected but not clearly identified. CEA was 238.0 ng/mL.
Diagnostic imaging	CT suggested invasion of the posterior vaginal wall.
Pretreatment	Double-barrel transverse colostomy was created.
CCRT	Capecitabine 1800 mg/day with pelvic irradiation of 50.4 Gy in 28 fractions.
Post-CCRT therapy	Three cycles of capecitabine maintenance therapy; CEA decreased to 3.3 ng/mL.
Restaging	Endoscopy showed marked tumor regression; imaging suggested RVF.
Preoperative assessment	The vagina was narrow and contracted after irradiation; direct transvaginal fistula manipulation was difficult.
Surgery, 12 weeks after CCRT	Laparoscopic bladder-preserving posterior pelvic exenteration was performed.
Intraoperative findings	The Douglas pouch and rectovaginal plane were obliterated by dense fibrosis and adhesions.
Surgical procedure	En bloc resection of the rectum, uterus, and posterior vaginal wall was completed. Operative time was 6 h 23 min; blood loss was 334 g.
POD 5	CRP increased to 24.7 mg/dL; cefmetazole was started for suspected pelvic infection.
POD 10	Fever developed; CRP was 13.2 mg/dL. CT confirmed pelvic peritonitis, and antibiotics were escalated to tazobactam/piperacillin.
After POD 10	Antibiotics were subsequently changed to levofloxacin 500 mg/day after 5 days.
POD 21	Discharged after clinical improvement.
Pathology	Well-differentiated adenocarcinoma; treatment response grade 2; ypT1bN0, ypStage I.
Follow-up, 3 months	No recurrence or procedure-related complications; PS remained unchanged.

CCRT, concurrent chemoradiotherapy; CRP, C-reactive protein; CT, computed tomography; and PS, performance status

Restaging endoscopy showed marked tumor regression (Supplemental Digital Content Figure S2a, available at: https://links.lww.com/IJSCR/A179). Post-treatment MRI demonstrated a decrease in tumor size but persistent suspected involvement of the vaginal wall and a visible RVF (Supplemental Digital Content Figure S2b, available at: https://links.lww.com/IJSCR/A179). Hysterography, barium enema, and vaginal cytology were not performed; the diagnosis of RVF and assessment of residual local extension were based on pelvic examination, endoscopic findings, and MRI. Laparoscopic bladder-preserving posterior pelvic exenteration was performed 12 weeks after completion of chemoradiotherapy. Preoperatively, direct visualization and manipulation of the fistula during speculum examination were difficult because of the narrow vaginal canal, tumor-related vaginal wall involvement, and post-radiation vaginal contracture.

Intraoperatively, dense fibrosis and adhesions around the rectum, cervix, and posterior vaginal wall obliterated the Douglas pouch and rectovaginal plane, making conventional posterior dissection via the abdominal approach unsafe. After the gynecologic team initiated the hysterectomy, vaginal-side retrograde resection of the posterior vaginal wall was performed to obtain access to the rectovaginal space. A posterior vaginal wall incision was made caudal to the fistula tract to avoid direct disruption of the fistula (Fig. 1A), thereby creating a controlled entry point into the rectovaginal space (Fig. 1B). The uterosacral ligaments and posterior vaginal wall were then transected en bloc with the rectum.

**Figure 1.**
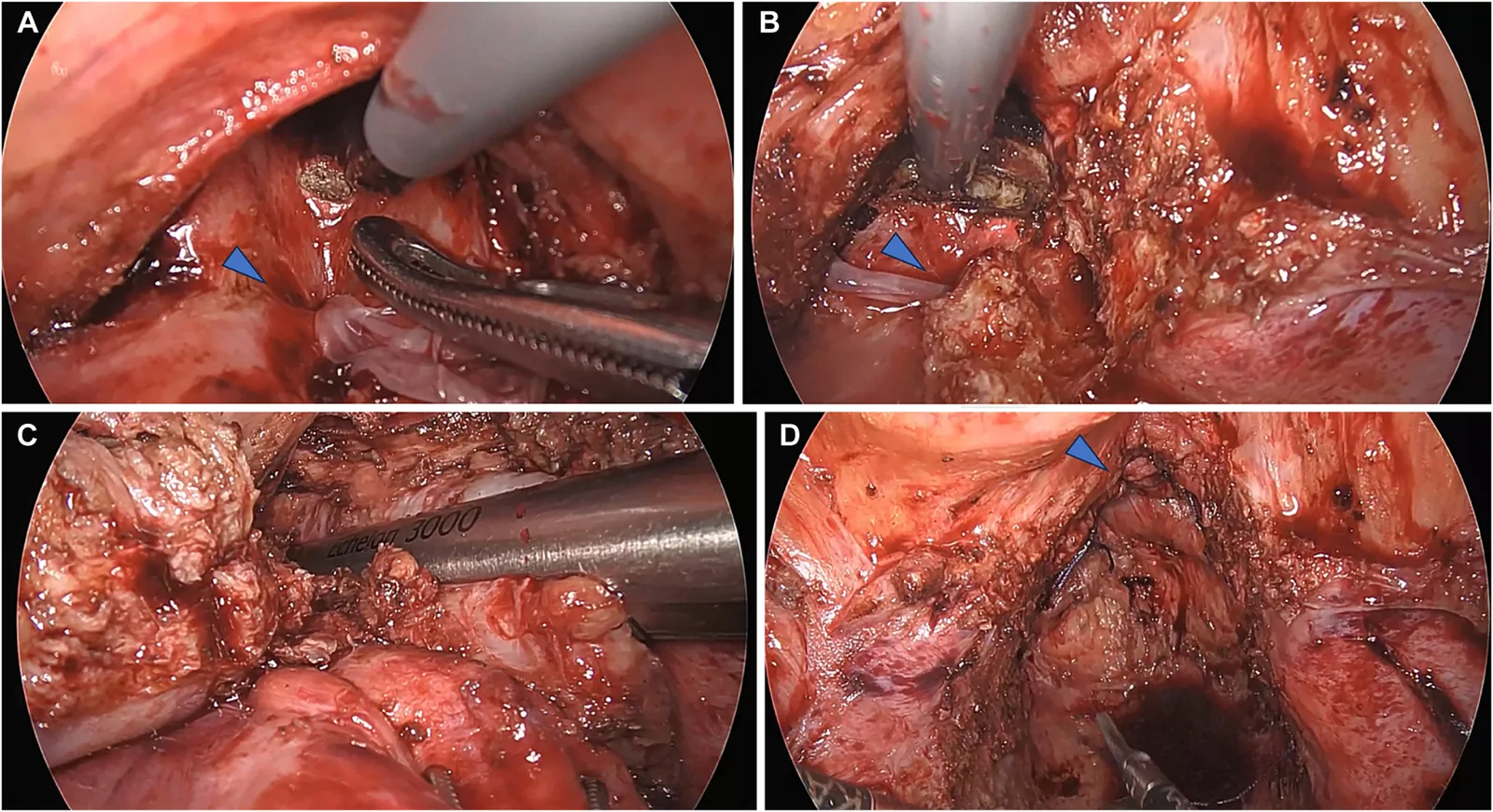
Laparoscopic surgical findings. (A) An incision is made in the posterior vaginal mucosa, caudal to the fistula located on the posterior vaginal wall (blue arrowhead). (B) The caudal incision relative to the fistula (blue arrowhead) is extended dorsally to enter the rectovaginal space. (C) The lower rectum is transected using an automatic stapling device. (D) After en bloc resection of the rectum, uterus, and posterior vaginal wall, the vaginal stump is closed with sutures (blue arrowhead).

The colorectal team completed a rectal resection and a Hartmann procedure (Fig. 1C). The rectum, uterus, and posterior vaginal wall were removed en bloc without tumor violation (Supplemental Digital Content Figure S3, available at: https://links.lww.com/IJSCR/A179). The vaginal stump was closed with sutures (Fig. 1D), and a pelvic drain was placed. The operative time was 6 hours and 23 minutes, and the estimated blood loss was 334 g.

Postoperatively, the patient was managed in the intensive care unit and received four units of red blood cells. She was transferred to the general ward on postoperative day (POD) 1, and oral intake was resumed on POD 4. On POD 5, the C-reactive protein level increased to 24.7 mg/dL, and cefmetazole (1 g/day) was initiated as empirical therapy for suspected pelvic infection. On POD 10, she developed a fever, with a C-reactive protein level of 13.2 mg/dL, and computed tomography showed findings consistent with pelvic peritonitis. No drainable abscess or fluid collection was identified; therefore, peritoneal aspiration and bacterial culture were not performed. Blood cultures were negative. Because the fever and inflammatory findings persisted, antibiotics were escalated to piperacillin–tazobactam, followed by levofloxacin (500 mg/day) after 5 days. The inflammatory response gradually resolved, and the patient was discharged on POD 21. The postoperative complications were classified as Clavien–Dindo grade II because they were managed with antibiotics and blood transfusion without invasive drainage or reoperation.

Histopathological examination revealed a residual well-differentiated adenocarcinoma limited to the rectal submucosal layer. Although pretreatment MRI suggested direct invasion of the posterior vaginal wall and uterine, the final pathological findings indicated marked downstaging post-chemoradiotherapy, with no viable tumor cells identified in the vagina or uterus. The RVF was identified in the specimen as a fistulous tract between the rectum and vagina, accompanied by treatment-related fibrosis and inflammatory changes around the tract and in the adjacent gynecologic tissues. The pathological response to neoadjuvant therapy was grade 2, and the final pathological stage was ypT1bN0, ypStage I. All resection margins were negative. Molecular analysis showed rat sarcoma viral oncogene homolog mutation positive, B-Raf proto-oncogene, serine/threonine kinase wild-type, microsatellite stable, and human epidermal growth factor receptor 2 negative. At the 10-month follow-up, there was no evidence of recurrence or subsequent procedure-related complications, and the patient’s performance status remained unchanged.

## Discussion

The key concept of this case is the use of a gynecologic–colorectal collaborative access strategy for a severely fibrotic pelvis. Dense adhesions obliterated the Douglas pouch and rectovaginal plane, while vaginal narrowing and post-radiation contracture limited conventional transvaginal manipulation. Although the final pathology showed marked downstaging to ypT1bN0, post-treatment MRI demonstrated persistent vaginal wall involvement and RVF, making residual tumor invasion difficult to exclude. Therefore, en bloc resection of the rectum, uterus, and posterior vaginal wall was performed to avoid tumor violation and achieve an R0 margin.

The novelty of this case is not posterior colpotomy itself, as retrograde hysterectomy and posterior vaginal wall incision have been described in gynecologic surgery and oncology^[^12,13^]^. Rather, this report adapts a gynecologic access concept to colorectal surgery as a practical retrograde entry strategy when abdominal access to the rectovaginal space is unsafe due to severe post-treatment fibrosis.

Several strategies reduce contamination or tumor cell spillage during vaginal transection^[^1,11,15–17^]^. Kawada *et al* described distal vaginal cuff closure before transection^[^16^]^. Yang *et al* reported laparoscopic total pelvic exenteration using a fascial space priority approach^[^17^]^. However, these approaches may require more extensive vaginal mobilization. In the present case, total pelvic exenteration or circumferential vaginal cuff closure was considered excessively invasive because the patient was 85 years old, had no clinical evidence of bladder involvement, and had severe vaginal narrowing and post-radiation contracture. Therefore, bladder-preserving posterior pelvic exenteration with limited vaginal-side retrograde access was selected.

However, this approach carries risks. In patients with RVF, vaginal lumen exposure may increase bacterial contamination or tumor cell spillage^[^9,18^]^. Accordingly, this technique should not be considered standard for T4b rectal cancer with RVF. Its key principle is controlled entry into the rectovaginal space without direct disruption of the fistula tract, followed by en bloc resection.

Postoperative pelvic peritonitis was managed with antibiotics without drainage or reoperation. Its etiology remained unclear and could not be attributed solely to exposure of the vaginal lumen. Because peritoneal cultures were not obtained and blood cultures were negative, the causative organism and infection source could not be identified. Possible contributing factors included the pre-existing RVF, prior pelvic irradiation, extensive adhesiolysis, and pelvic dead space^[^18–21^]^.

Potential candidates for this approach may include patients with severe post-treatment fibrosis obscuring the pouch of Douglas and the rectovaginal plane, persistent RVF requiring en bloc resection, restricted vaginal access, no bladder involvement, and situations in which total pelvic exenteration would be excessively invasive.

This report is limited by its single-case design and short follow-up. No recurrence was observed at 10 months; however, longer follow-up is needed to assess oncologic safety, fistula-related recurrence, functional outcomes, and quality of life. Although formal quality-of-life assessment was not performed, the patient had no subsequent procedure-related complications and her performance status remained unchanged. This approach requires close collaboration between colorectal and gynecologic surgeons and should be reserved for carefully selected patients at centers with appropriate expertise^[^22^]^. Further experience is needed to clarify its indications and limitations.

## Conclusion

This case demonstrates a gynecologic–colorectal collaborative vaginal-side retrograde approach for laparoscopic bladder-preserving posterior pelvic exenteration in a severely fibrotic pelvis. When conventional abdominal entry into the rectovaginal space is unsafe, retrograde posterior vaginal wall resection may provide controlled access for en bloc resection.

This approach should be reserved for carefully selected patients, as the evidence is limited to a single case. Further experience and longer follow-up are required to define its indications, risks, and oncologic safety.

## Data Availability

The clinical data that support the findings of this case report are not publicly available due to concerns regarding patient privacy, in accordance with institutional and ethical guidelines.
